# New technique for false lumen coiling of spontaneous isolated superior mesenteric artery dissection

**DOI:** 10.1186/s42155-021-00225-7

**Published:** 2021-04-07

**Authors:** Hidenori Yamaguchi, Satoru Murata, Tatsuo Ueda, Takahiko Mine, Shiro Onozawa, Hiromitsu Hayashi, Shin-ichiro Kumita

**Affiliations:** 1 Department of Radiology, Nippon Medical School, Tamanagayama Hospital, 1-7-1, Nagayama, Tama-shi, Tokyo, 206-8512 Japan; 2 Center for Interventional Radiology, Teikyo University Chiba Medical Center, 426-3 Anesaki, Ichihara-City, Chiba, 299-0011 Japan; 3 Department of Radiology, Nippon Medical School, 1-1-5 Sendagi,Bunkyo-ku, Tokyo, 113-8603 Japan; 4 Department of Radiology, Nippon Medical School, Chibahokusoh Hospital, 1715 Kamagari, Inzai, Chiba, 270-1694 Japan; 5 Department of Radiology, Kyorin University Hospital, 2-60-2 Shinkawa, Mitaka-City, Tokyo, 181-8611 Japan

**Keywords:** Dissection, Mesenteric artery, Endovascular intervention, Coil, Embolization

## Abstract

**Background:**

Spontaneous isolated visceral artery dissection is rarely encountered. Endovascular intervention with good outcomes has become popular for patients with persistent symptoms or developing ischemia. We could perform life-saving treatment for a spontaneous isolated superior mesenteric artery dissection with a unique endovascular intervention.

**Case presentation:**

We describe the case of an 80-year-old man who presented with acute abdominal pain and a spontaneous isolated superior mesenteric artery dissection measuring 35 mm in major diameter and 6.6 mm in minor diameter on abdominal contrast-enhanced computed tomography. After admission, abdominal pain was progressive, and a repeated scan revealed progression of the dissection. As an endovascular intervention, via the bilateral femoral approach, detachable coils were placed in the false lumen of the superior mesenteric artery dissection through the false lumen under the micro-balloon occlusion at the point of re-entry and entry through the true lumen to prevent coil migration. Technical and clinical success was achieved without serious adverse events.

**Conclusion:**

Coil embolization using micro-balloon assistance combined with the double-catheter technique for a large entry and re-entry false lumen of a spontaneous isolated superior mesenteric artery dissection was useful and feasible.

## Background

Spontaneous isolated visceral artery dissection (SIVAD), first described in 1947 (Bauersfeld 1947), has an incidence of 0.68% among all abdominal contrast-enhanced computed tomography (CE-CT) scans taken for acute abdominal symptoms (Yamaguchi et al. 2019). Presumed risk factors include atherosclerotic disease, hypertension, fibromuscular dysplasia, cystic medial necrosis, and connective tissue disorders (Yamaguchi et al. 2019; Takayama et al. 2008). Treatment options for SIVAD include nonoperative, endovascular, and surgical interventions (Yamaguchi et al. 2019; Pang et al. 2013; Alcantara et al. 2015; Sosogi et al. 2019; D'Ambrosio et al. 2007). Recently, appropriate treatment strategies have been proposed (Yamaguchi et al. 2019; Sosogi et al. 2019). Endovascular intervention with good outcomes has become popular for patients with persistent symptoms or developing ischemia because of comparable outcomes with surgical intervention (Yamaguchi et al. 2019; Takayama et al. 2008; Pang et al. 2013). We report about a spontaneous isolated superior mesenteric artery (SMA) dissection of performing life-saving treatment with a unique endovascular intervention.

## Case presentation

**Fig. 1:**
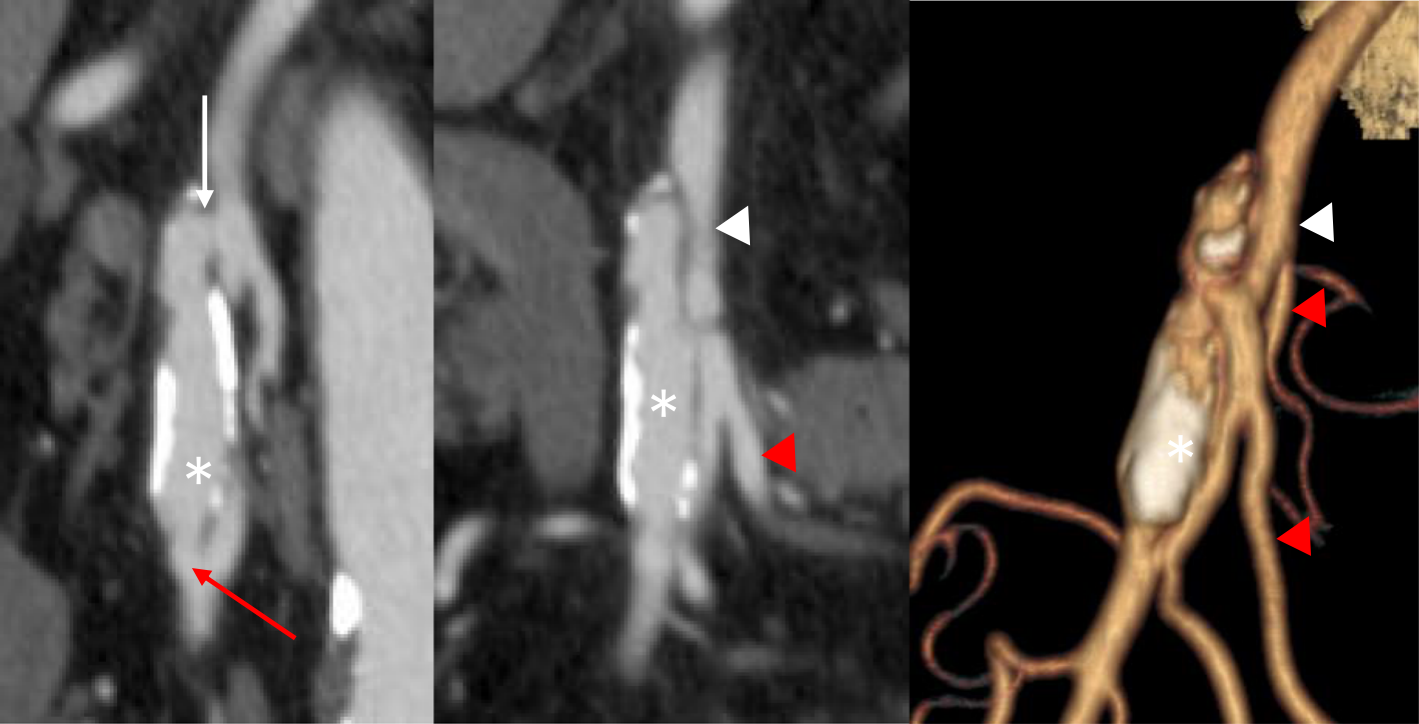
Abdominal contrast-enhanced computed tomography (CT) scans. Multiplanar reconstruction and 3-dimensional CT showed the spontaneous isolated superior mesenteric artery dissection measuring 35 × 6.6 mm in diameter. *: false lumen; white arrow: entry; red arrow: re-entry; white arrowhead: true lumen; red arrowhead: jejunal artery

An 80-year-old man with a history of hypertension, atrial fibrillation, and diabetes mellitus presented with severe abdominal pain, distention, and tenderness. Spontaneous isolated SMA dissection was diagnosed based on abdominal CE-CT and measured 35 mm in major diameter and 6.6 mm in minor diameter with a large entry and re-entry (Fig. 1). Vital signs were as follows: systolic blood pressure, 184 mmHg; respiratory rate, 22 breaths/min; heart rate, 124 beats/minute; and oxygen saturation via pulse oximetry, 100% with room air. Hemodynamic parameters were stable. After admission, abdominal pain worsened, and CE-CT revealed progression of the dissection.

**Fig. 2:**
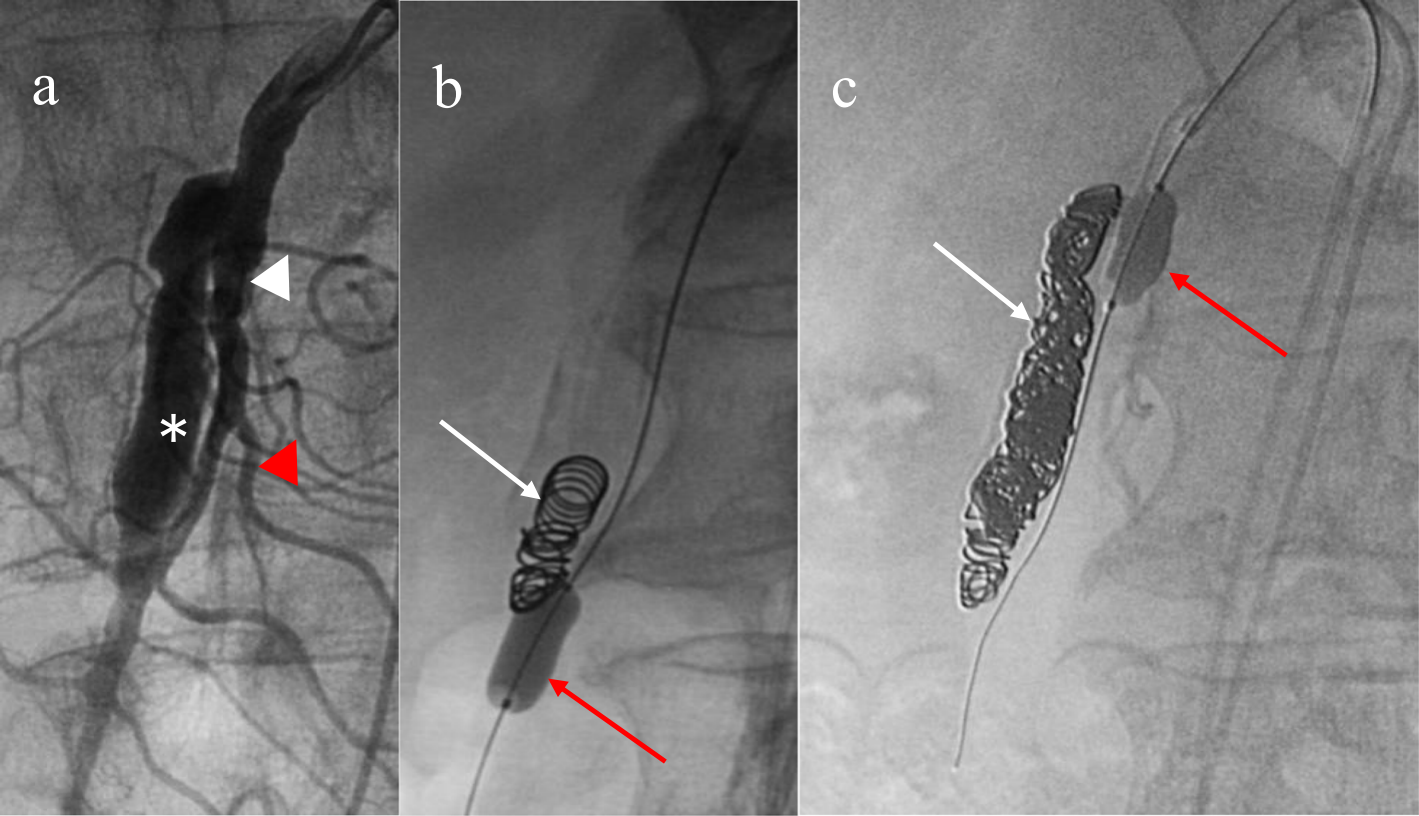
Digital subtraction angiography of the superior mesenteric artery (SMA). **a** Superior mesenteric arteriography demonstrated a dissection originating in the SMA and involving the jejunal arteries. **b** The re-entry through the true lumen was occluded with the micro-balloon to prevent coil migration. Using a 2-marker catheter through the false lumen, eight detachable coils were placed in the false lumen. **c** The micro-balloon was slowly pulled to the point of entry and re-expanded, and three detachable coils were placed in the false lumen. *: false lumen; white arrowhead: true lumen; red arrowhead: jejunal artery; white arrow: coils; red arrow: micro-balloon

**Fig. 3:**
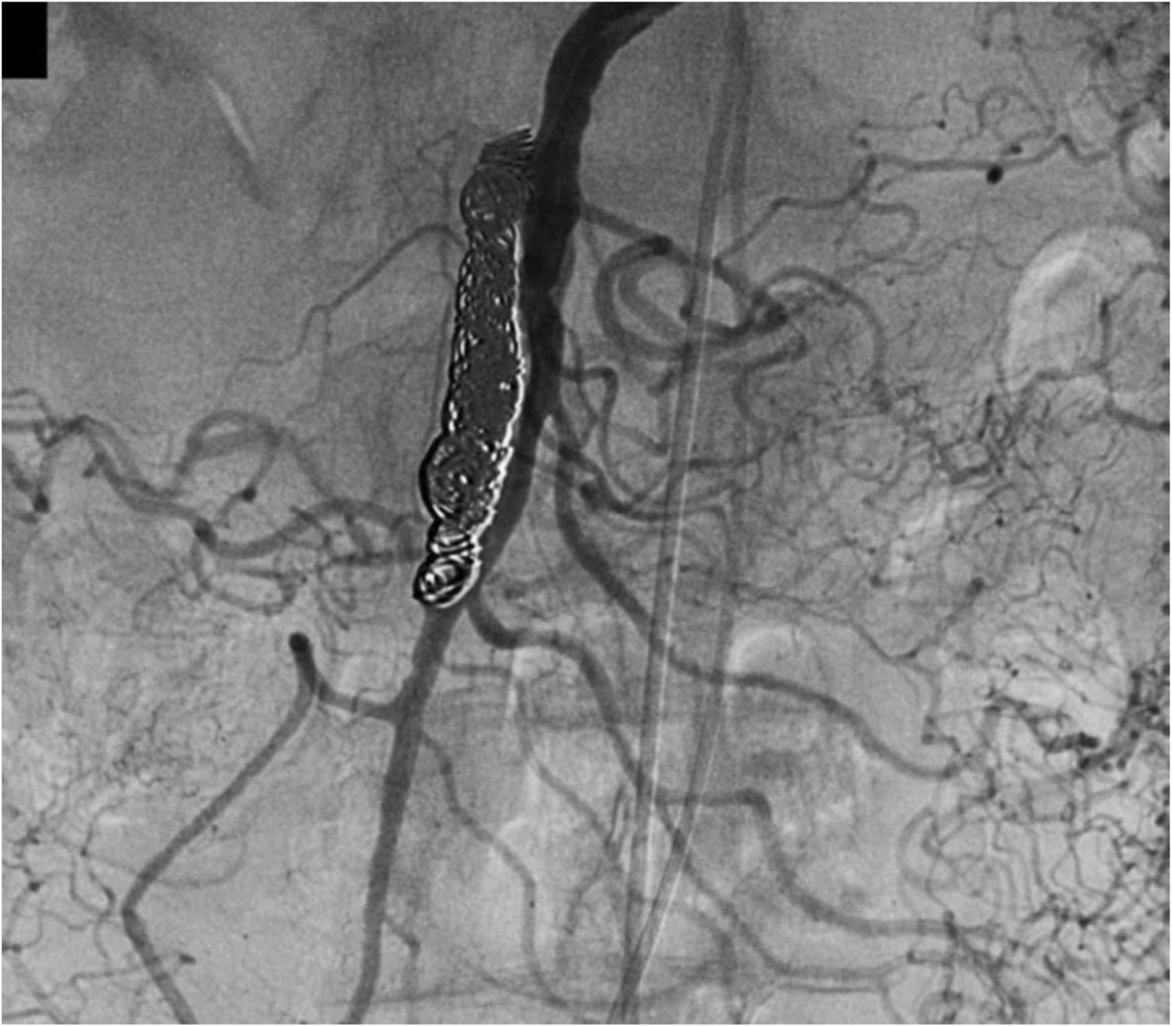
After coil embolization, superior mesenteric arteriography showed disappearance of the dissection with blood flow in the superior mesenteric artery and the jejunal arteries without perfusion delay

Endovascular intervention was performed under local anesthesia. Superior mesenteric arteriography using the right femoral approach through a 4-French catheter (Shepherd; Medikit Co. Ltd., Miyazaki, Japan) demonstrated a dissection originating in the SMA and involving the jejunal arteries (Fig. 2a). The dissection measured 35 mm in major diameter and 6.6 mm in minor diameter, with a large entry and re-entry. We judged that coil embolization of the false lumen was suitable. However, blood flow was considerable, and it was impossible to stabilize the coils. Accordingly, an approach was taken via the bilateral femoral arteries with 4-French systems to embolize the false lumen using a 1.8-French micro-balloon (LOGOS®; PIOLAX, Inc., Yokohama, Japan) and the double-catheter technique. First, the re-entry through the true lumen was occluded with the micro-balloon to prevent coil migration (Fig. 2b). Second, using a 2.2-French 2-marker catheter (Coiling Support; HI-LEX MEDICAL®, Hyogo, Japan), eight detachable coils (3 pieces, 4 mm × 10 cm; 3 pieces, 3 mm × 8 cm; 2 pieces, 2.5 mm × 5 cm; GALAXY G3™, Johnson & Johnson, New Jersey, USA) were placed in the false lumen. The micro-balloon was contracted, and the stability of the coils was confirmed. Finally, the micro-balloon was slowly pulled to the point of entry and re-expanded, and three detachable coils (3 pieces, 3 mm × 8 cm) were placed in the false lumen (Fig. 2c). Arteriography showed disappearance of the dissection with blood flow in the SMA and jejunal arteries without perfusion delay (Fig. 3).

On postoperative day 2, the abdominal pain resolved. The post-treatment course was favorable, and the patient was discharged on postoperative day 5.

## Discussion

Yamaguchi et al. (Yamaguchi et al. 2019) reported that SIVAD occurred in 0.68% of all abdominal CE-CT scans taken for acute abdominal symptoms. This suggests that SIVAD is rare, and consensus regarding the pathology and optimal therapy is lacking. However, some recent papers have advocated treatment strategies (Yamaguchi et al. 2019; Sosogi et al. 2019). Pathogenesis is unknown, except for Ehlers-Danlos syndrome, segmental arterial mediolysis, and trauma. Some investigators have associated SIVAD with hypertension and atherosclerosis (D'Ambrosio et al. 2007). Hypertension may be a predisposing factor; however, no data support its role in causing intimal tear. In cases of celiac artery (CA) stenosis or occlusion by arteriosclerosis and the median arcuate ligament, the compensatory increase in flow in the SMA may lead to increased shear stress (Takayama et al. 2008; Jung et al. 2013), leading to dissection. However, CA stenosis was not found in this patient. Anatomically, the point 10.0–30.0 mm from the SMA orifice, between the fixed retropancreatic portion and the mobile portion, is weak (Pang et al. 2013). In this patient, the distance from the SMA orifice to the intimal flap was 25 mm, which corresponds to this weak point. This suggests that anatomical weakness is significantly involved in pathogenesis.

Yamaguchi et al. (Yamaguchi et al. 2019) proposed that important signs in symptomatic SIVAD and changes in the CE-CT scan were associated with symptoms of ongoing bowel ischemia. On this basis, endovascular intervention was performed in this patient due to evidence of dissection progression.

Optimal treatment has not been established; however, endovascular intervention has become popular for patients with persistent ischemic symptoms, and outcomes are comparable to those of surgical intervention (Yamaguchi et al. 2019; Takayama et al. 2008; Pang et al. 2013; Jung et al. 2013; Park et al. 2011; Sakamoto et al. 2007). Therefore, endovascular therapy has become the first choice at our institution because of its minimal invasiveness.

To achieve successful endovascular intervention, the following criteria were considered. The dissection originated in the SMA and involved the jejunal arteries. The dissection entry and re-entry were large in diameter. Therefore, we speculated that it would be difficult to reduce blood flow in the false lumen even if a bare stent was implanted. In addition, covered stent placement for dissection is unsuitable in a sharply curved vessel and peripheral vessel, and long-term patency is poor. Furthermore, covered stent placement will unnecessarily occlude branch vessels. When performing coil embolization, maintaining coil stability is difficult because of rapid blood flow. In the case of peripheral coil migration, organ ischemia or necrosis is possible. Accordingly, flow control and coil stability are important for complete coil embolization. Therefore, we performed a unique coil embolization of the false lumen using micro-balloon assistance combined with the double-catheter technique, occluding the points of entry and re-entry and establishing coil stability.

## Conclusion

Coil embolization using micro-balloon assistance combined with the double-catheter technique for a large entry and re-entry false lumen of a spontaneous isolated SMA dissection was minimal invasiveness, useful, and feasible. This technique can be applied to every vessel dissection in hesitation of a covered stent placement.

## Data Availability

Not applicable.

## Abbreviations

SIVAD: Spontaneous isolated visceral artery dissection
CE-CT: Contrast-enhanced computed tomography
SMA: Superior mesenteric artery
CA: Celiac artery
